# A lifecycle framework illustrates eight stages necessary for realizing the benefits of patient-centered clinical decision support

**DOI:** 10.1093/jamia/ocad122

**Published:** 2023-07-06

**Authors:** Dean F Sittig, Aziz Boxwala, Adam Wright, Courtney Zott, Priyanka Desai, Rina Dhopeshwarkar, James Swiger, Edwin A Lomotan, Angela Dobes, Prashila Dullabh

**Affiliations:** McWilliams School of Biomedical Informatics, University of Texas Health Science Center at Houston, Houston, Texas, USA; Elimu Informatics, El Cerrito, California, USA; Department of Biomedical Informatics, Vanderbilt University Medical Center, Nashville, Tennessee, USA; NORC at the University of Chicago, Bethesda, Maryland, USA; NORC at the University of Chicago, Bethesda, Maryland, USA; NORC at the University of Chicago, Bethesda, Maryland, USA; Center for Evidence and Practice Improvement, Agency for Healthcare Research and Quality, Rockville, Maryland, USA; Center for Evidence and Practice Improvement, Agency for Healthcare Research and Quality, Rockville, Maryland, USA; Crohn’s & Colitis Foundation, New York, New York, USA; NORC at the University of Chicago, Bethesda, Maryland, USA

**Keywords:** clinical decision support, patient-centered care, shared decision making, medical informatics applications

## Abstract

The design, development, implementation, use, and evaluation of high-quality, patient-centered clinical decision support (PC CDS) is necessary if we are to achieve the quintuple aim in healthcare. We developed a PC CDS lifecycle framework to promote a common understanding and language for communication among researchers, patients, clinicians, and policymakers. The framework puts the patient, and/or their caregiver at the center and illustrates how they are involved in all the following stages: Computable Clinical Knowledge, Patient-specific Inference, Information Delivery, Clinical Decision, Patient Behaviors, Health Outcomes, Aggregate Data, and patient-centered outcomes research (PCOR) Evidence. Using this idealized framework reminds key stakeholders that developing, deploying, and evaluating PC-CDS is a complex, sociotechnical challenge that requires consideration of all 8 stages. In addition, we need to ensure that patients, their caregivers, and the clinicians caring for them are explicitly involved at each stage to help us achieve the quintuple aim.

## INTRODUCTION

Since 2016, and furthered with the 2019 renewal of the Patient-Centered Outcomes Research (PCOR) Trust fund,^1^ the Agency for Healthcare Research and Quality (AHRQ) has continuously focused on the legislative requirement to “Support the incorporation of research findings into health information technologies associated with clinical decision support.”^2^ The need to develop methods, tools, and frameworks to help healthcare organizations realize the benefits of patient-centered clinical decision support (PC CDS), for example, the need for increased patient engagement,^3^ has become more important. This article provides a comprehensive, idealized framework to delineate the PC CDS lifecycle and explore the nature of potential patient and/or caregiver participation at each stage of the process.

PC CDS, a subset of the clinical decision support (CDS) domain, focuses on CDS that is: (1) based on findings from PCOR; (2) utilizes patient-generated health data, patient-reported outcomes, social determinants of health (SDOH) data, and/or patient preference data; or (3) is delivered directly to patients, their caregivers, or healthcare providers to encourage shared decision-making.^4^ Designing, developing, implementing, using, and evaluating PC CDS is a complex, sociotechnical task that should involve patients, as much as possible, at every stage.

## DEVELOPMENT OF THE PC CDS FRAMEWORK

The PC CDS framework is based on a review of the literature and the experience of the authors. The authors have extensive knowledge, education, training, and experience gained from previous work on CDS projects and participation in national [DFS, AB, AW, EAL, JS, PDu], and international [DFS, AW, EAL] groups focused on setting policies and improving our understanding of CDS. The authors are experienced in healthcare informatics [all], clinical medicine [AB, EAL, PDu], health IT development [DFS, AW, JS, EAL, AB, AD], patient-centered outcomes research [DFS, AB, AW, AD, PDe, PDu], patient engagement [DFS, AD, PDu], and patient advocacy [AD]. All authors participated in several virtual meetings to discuss findings and synthesize the framework. Meeting minutes were reviewed and used to iteratively refine the framework. Finally, the team vetted the framework with a 7-member technical expert panel (see “Acknowledgments”), the AHRQ-funded Clinical Decision Support Innovation Collaborative (CDSiC) project steering committee,^5^ and the CDSiC workgroups which included patients and patient advocates.

**Figure 1.**
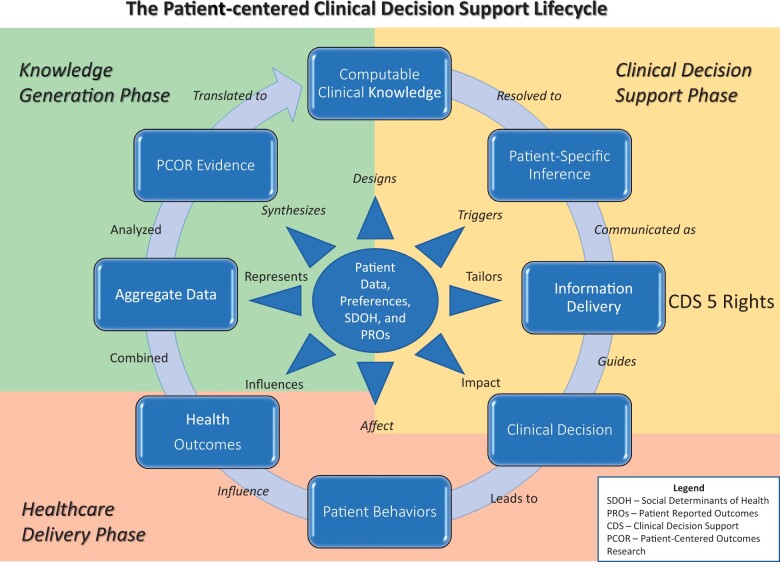
Diagram illustrating the various steps, their sequence, and relationships necessary to design, develop, implement, use, and manage patient-centered clinical decision support across its lifecycle.

The idealized PC CDS lifecycle framework (see Figure 1) combines, extends, and refines elements and relationships from 4 existing theoretical frameworks:

1. **CDS Five-Rights,** which describes how CDS interventions should ensure the right information is delivered to the right person, in the right format, via the right channel, at the right point in the workflow.^6^
2. **Multilayer Knowledge Representation Framework,** which describes how guidelines can be increasingly structured from narrative text to computable knowledge^7^—most importantly, by incrementally separating the clinical knowledge in the recommendation from the operational knowledge required to implement a CDS recommendation within the application.
3. **Learning Health System,** which describes a virtuous lifecycle in which healthcare organizations efficiently and effectively deliver high-quality, evidence-based care while simultaneously producing new clinical and operational knowledge. This new knowledge can then be incorporated via an electronic health record (EHR) application, for example, back into the care delivery processes.^8^
4. **Analytical Framework for Action,** which describes 4 components for disseminating evidence models^9^ to help describe the phases, relationships, sequences, and interacting activities necessary for the design, development, implementation, use, and evaluation of PC CDS.

These 4 frameworks were selected from a much larger group of CDS-related models and frameworks^10^ based on their importance, widespread use, inclusion of patient-centered components, and focus on those aspects of CDS that we believe are most important to help us identify new opportunities and challenges facing PC CDS. The article’s recommendations for patient engagement within each lifecycle stage will be difficult to achieve. But for this idealized description of the PC CDS lifecycle to be realized, it is crucial to center the effort throughout on patient-centered options for meaningful patient engagement.

## THE PATIENT-CENTERED CLINICAL DECISION SUPPORT LIFECYCLE FRAMEWORK

At the highest level, the PC CDS lifecycle 8-stage framework consists of 3 overarching phases: Knowledge Generation, Clinical Decision Support, and Healthcare Delivery. Briefly, the *Knowledge Generation* phase includes developing evidence and creating guidelines, based on patient-centered outcomes research (PCOR),^11^ which involves patient engagement throughout the research process to ensure that the research accounts for the needs and preferences of patients. PCOR can also include observational studies that analyze EHR data from large numbers of patients to develop the evidence base necessary to answer specific healthcare and lifestyle-related clinical questions.^12^ The goal of this research is to address the quintuple aims of improving population health, enhancing patients’ care experience, reducing costs, reducing burnout among healthcare workers, and advancing health equity.^13–15^ PCOR research also addresses the National Academy of Medicine’s quality targets of providing safe, timely, effective, efficient, equitable, and patient-centered care.^16^

The *Clinical Decision Support* phase encompasses the transformation of evidence from clinical research studies, expert opinion, and analysis of clinical practice patterns into computable logic that can be used to generate and deliver trusted, high-quality, accurate, timely, reliable, comprehensible, patient-specific recommendations to patients, their caregivers, care teams,^17^ or their clinicians.^18^ Over the years there have been many attempts to develop such computable logic in forms and formats that are able to be shared across organizations with limited success.^19^ The recommendations that result from this computable logic should meet the 5 rights of CDS.^20^

The *Healthcare Delivery* phase embodies the clinical decision-making process. This decision-making process is influenced by specific knowledge and understanding of the clinician, patient, and caregiver. It also depends on patients’ and clinicians’ beliefs, lived experiences, and any organizational or personal constraints or limitations.

**Figure 2.**
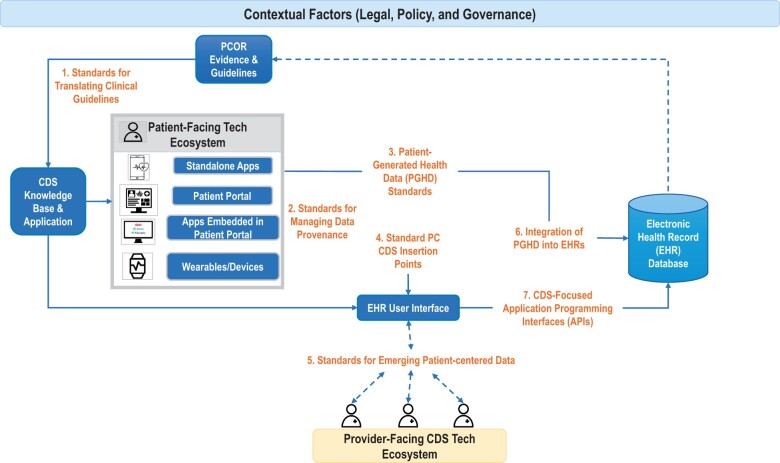
Diagram showing the standards involved in presenting clinical decision support recommendations to patients and in collecting their responses and outcomes in the EHR. Used with permission from: J Am Med Inform Assoc. 2022 May 11;29(6):1101-1105. doi: 10.1093/jamia/ocac029.

The degree to which these elements are incorporated into evidence-based, clinical care decisions affects the likelihood that the advice will be followed. The decision of what, when, where, and how to carry out each recommendation is ideally made by the patient in consultation with their caregiver(s) and care team(s). In emergencies, or when the patient is unable to participate in the decision and their caregivers are not available, the decision may be made entirely by their clinician(s). These decisions and the resulting behaviors lead to health outcomes. In the best of all possible worlds, these clinical decisions, patient behaviors, and health outcomes would be accurately captured, promptly, at the right time, in a standard, structured form within an EHR so they can activate PC CDS logic statements (see Figure 2).^21^

Within the PC CDS lifecycle framework, we place the patient, combined with all the contextual data required to describe their physiological, psychological, economic, and social conditions and preferences, at the center.^22^ These patient data are used in different ways in each of the 8 stages and 3 phases of the lifecycle, as indicated by the verbs (eg, triggers, tailors, or influences) emanating from the inner circle that includes examples of the types of patient data available (eg, patient preferences or SDOH). For example, in the knowledge generation phase, patient data from EHRs and other sources provide the basis for the evidence that is developed. In the *clinical decision support* phase, patient-generated health data drive patient-centric CDS recommendations, while in the *healthcare delivery* phase, patient data influence the healthcare outcomes experienced.

Within these 3 overarching phases, we have identified 8 specific stages of the PC CDS lifecycle. Each stage must be successfully navigated, with as much patient and/or caregiver input as possible and with as little information loss as possible, if the PC CDS intervention is going to be effective in achieving the quintuple aim. The following sections describe each of these stages in detail. Each section focuses on the work that needs to be completed and the data that must be collected, analyzed, transmitted, or displayed. Each section also highlights the potential role of the patient and/or their caregiver (hereafter referred to as patients) in completing this work. Their contributions can greatly aid researchers, PC CDS developers, and clinicians in accomplishing these tasks.

### Computable clinical knowledge

The clinical knowledge transformation process begins following agreements among guideline developers, patients, clinicians, health policymakers, informaticians, and payors who collaboratively determine which clinical evidence can and should be translated into guidelines and/or PC CDS interventions. Transforming PCOR evidence into computable clinical knowledge requires the skill and experience of a clinical informatician,^23^ working with clinicians or guideline developers.^24^ This person must be steeped in the science of clinical knowledge management and capable of extracting and encoding sound clinical logic from often ambiguous clinical evidence or even guidelines; identifying structured, patient-specific data items that can be accurately recorded; and mapping data and recommendations to existing structured, clinical vocabularies.^25^ Patient and caregiver contributions at this stage can occur in 3 important ways. First, they can provide input into what guidelines are prioritized for translation into PC CDS so that the PC CDS is designed to achieve health outcomes that are not just clinically desirable but also meaningful for patients. Second, patients can collaborate on how guidelines are translated to PC CDS, to build trust and transparency in this process. Third, patients can work with PC CDS developers to agree on what data will be collected, when they will provide this data, and how the data to drive the PC CDS will be collected. A layered approach to translating evidence or guidelines into computable CDS knowledge that takes into consideration each stakeholder’s clinical and technical capabilities can facilitate the incorporation of patients’ needs and preferences.^7^

### Patient-specific inference

Patient-specific clinical advice is generated when new patient-specific data, the passage of time, or an event triggers computable clinical knowledge which is then resolved to create the patient-specific inference.^26^ For example, a recommendation could be triggered when the patient’s weight or blood pressure is captured, the patient turns 60 years old, 6 months have passed since the patient’s last HbA1c measurement, or when the patient arrives at the clinic, or gets discharged from the hospital. A potential role for patients at this stage is to work with PC CDS developers to: (1) codesign digital tools to capture the required data elements; and (2) deliver the clinical advice in a format that presents an easy-to-understand rationale based on sound clinical evidence.^27^ Finally, the advice should be accessible to the patient (ie, easy to understand, unambiguous, and actionable).^28^ This patient-specific inference may occur directly in the EHR or in an external inference system integrated with the EHR using a standard such as CDS Hooks.^29^

### Information delivery

Satisfying the CDS Five Rights—communication of the *right information* to the *right people* in the *right formats*, via the *right channels*, at the *right points in the workflow* (to be interpreted in the PC CDS context as the workflow of the healthcare provider or *life flow* of the patient) is critical for success. Failure to properly account for, or achieve, any one of these “Five-rights” often results in the PC CDS failing to reach its intended audience, resulting in suboptimal clinical decision-making. A potential role for patients at this stage is to work with PC CDS developers to identify the options and help developers understand patient preferences for when, where, and how to deliver the information. In general, personalization is good. But when the CDS system uses information a patient may consider private or sensitive, extreme personalization can become intrusive and the advice rejected.^30^ Highly personalized PC CDS should provide transparency on the source of the information used to personalize the information, and provide a way for patients to opt-out of having their information used in this manner.^31^

### Clinical decision

A good clinical decision is guided by accurate, up-to-date patient-specific data, the most relevant clinical evidence, and the patient’s needs, desires, values, and abilities. While clinicians may not always agree with the patient’s decisions, except in the most extreme cases, the patients have the final say. Therefore, the goal of PC CDS should be to enhance the likelihood that the final decision is based on a shared decision that reflects an understanding and discussion of the patient’s needs, desires, and abilities that matter most.^32^ Healthcare providers must also be cognizant that the patient’s decision—especially in cases that involve long-term, repeated actions, like taking daily medication for a chronic condition—can change in the future. Thus, a provider must be willing and able to monitor the patient’s behavior (see further discussion in “Patient behaviors” below), and re-engage in patient-reported outcomes-based data collection and shared decision-making as necessary. One way for patients to become engaged at the clinical decision stage is for them to work to understand the evidence and reasoning behind the decision; clearly articulate their own needs, desires, and abilities; and work with their healthcare provider to facilitate informed health decisions and reach the best solution for everyone.^33^

### Patient behaviors

Clinical decisions lead to patient behaviors. In this stage, patients decide whether or not to take the specified actions. The effectiveness of their actions is based on a combination of the effectiveness and relevance of the suggested activities, previous clinical and lifestyle decisions, and the level of adherence to the action the patient is willing and able to achieve. To date, there is very little research, let alone evidence, to help identify keys for success in understanding how to close the gap between “clinically desirable” patient behaviors and outcomes that are important to patients.^34^ Often, the potential long-term benefits of clinical advice (eg, medications for chronic conditions) are outweighed by the negative short-term side effects.^35^ The entire healthcare delivery system struggles to capture the data required to measure, monitor, or in many cases, intervene when things are not going according to plan. A role for patients at this stage is to learn as much as they can about their illness, communicate with their healthcare providers when they experience problems, and answer questions or send physiological monitoring data if asked.

### Health outcomes

Patient-oriented health outcomes are influenced by factors that include the patient’s environmental, genetic, physiological, and social circumstances. The decisions and behaviors of patients, caregivers, and clinicians also play a role. Furthermore, these outcomes are influenced by the psychological and physiological effects of the interventions undertaken. In some circumstances, the patient’s preferences for specific outcomes may be different from those of their clinicians. For example, a patient might prioritize the ability to drive over pain management for some musculoskeletal disorders, or even quality over quantity of life. One way for patients to be engaged in the health outcome stage is for them to periodically report on specific aspects of their lives. These could include their physiological condition, psychological well-being, and living environment. They could also report their ongoing preferences for desired outcomes. In addition, patients can help clinicians understand the minimal clinically important difference in symptom management or outcomes that would change their behavior.^36^ It is important to note that these preferences may change as their conditions improve or deteriorate, thus necessitating adjustments in therapy.

### Aggregate data

To understand and interpret the causes of patients’ health outcomes, researchers need to combine comprehensive, longitudinal data from many patients to completely characterize their health, their healthcare experience, and the complex clinical context in which they live. These data rarely exist in a single EHR database. Therefore, epidemiology and health services researchers interested in aggregating data must search far and wide to gather, organize, and map the required longitudinal data into a common format.^37^ In many cases researchers settle for proxies (eg, zip codes to estimate income)^38^ or indirect indicators (eg, measures of body composition to predict certain diseases or hospitalization)^39^ to represent the more specific data they ideally need. In most cases, even an indicator as basic as whether a particular patient has died is seldom accurately and promptly recorded in a healthcare organization’s EHR.^40^ Patients and patient advocates can play a key role in at least 2 respects. First, they can help make patient data available for future quality improvement and research. Second, they can work with health systems and other organizations that hold patient data to build more clarity and transparency into what, and how, patient data are collected, shared, and used for research. The new rules restricting information blocking in the 21st Century Cures Act are certainly well intentioned. It remains to be seen what their impact will be.^41^^,^^42^

### PCOR evidence

PCOR is designed to analyze the benefits and harms of clinical care over a full range of clinical and patient-centered outcomes that are both meaningful to patients and delivered in real-world settings. PCOR generally compares 2 healthcare options, rather than the more traditional randomized clinical trial approach of comparing intervention and control groups. Patients can engage at this stage by bringing their lived experience to the condition being studied, challenges in navigating the healthcare system, and a willingness and ability to share these experiences to help influence the research at every stage—to make sure it is patient-centered, relevant, and useful to the widest range of patients.^43^

## POTENTIAL BARRIERS TO PATIENT PARTICIPATION

Despite the potential for overwhelming benefits associated with increased patient participation in the PC CDS life cycle, numerous and substantial barriers remain, on the part of both those representing the healthcare system and those on the patient side, that must be overcome. On the patient side, a seminal article by Longtin et al^44^ identified key factors that influence patient participation. These barriers include low health literacy^45^; lack of knowledge of the subject, confidence in their knowledge or ability; type of input required (eg, patients are better suited to decision-making situations that focus on the value or utility of different options, rather than solving specific problems that require specialized knowledge)^46^; condition or disease severity^47^; as well as age, sex, socio-economic status, educational level, ethnic and cultural differences, and lack of trust in the healthcare system.^48^ On the clinical and technical side, barriers include a desire to maintain control, lack of time to listen and respond to suggestions, personal experience, beliefs, and insufficient training on how to include patients in these complex processes.^44^ Significant progress must be made in addressing these complex, sociotechnical barriers if we are to achieve our goals.

## CONCLUSION

Collaborative design, development, implementation, use, and evaluation of high-quality PC CDS are all necessary if we are to achieve the quintuple aim of healthcare improvement. This article presents a PC CDS lifecycle framework developed to promote a common understanding and language for communication among researchers, patients, clinicians, and policymakers. This framework expands upon: (1) the CDS Five-rights model, which focuses on the Information Delivery stage; and (2) the Multilayer Knowledge Representation Framework, which focuses on the Computable Clinical Knowledge stage. This new framework also adds PC CDS-specific details to the Learning Health System model and illustrates the importance of patient input at each phase of the Analytical Framework for Action. Using this new framework will remind key stakeholders that developing, deploying, and evaluating PC CDS is a complex, sociotechnical challenge that requires consideration of at least the 8 stages identified in the framework. Using the framework will also ensure that patients, their caregivers, and the clinicians caring for them are included at each stage of the PC CDS lifecycle—essential, if we are to fully achieve the quintuple aim.

## Data Availability

Not applicable.
